# 7-*O*-Methylpunctatin, a Novel Homoisoflavonoid, Inhibits Phenotypic Switch of Human Arteriolar Smooth Muscle Cells

**DOI:** 10.3390/biom9110716

**Published:** 2019-11-08

**Authors:** Manal Fardoun, Rabah Iratni, Hassan Dehaini, Assaad Eid, Tarek Ghaddar, Tamam El-Elimat, Feras Alali, Adnan Badran, Ali H. Eid, Elias Baydoun

**Affiliations:** 1 Department of Biology, Faculty of Arts and Sciences, American University of Beirut, P.O. Box 11-0236, Beirut, Lebanon; mmf27@mail.aub.edu; 2 Department of Biology, College of Science, United Arab Emirates University, P.O. Box 15551, Al-Ain, UAE; r_iratni@uaeu.ac.ae; 3 Department of Pharmacology and Toxicology, Faculty of Medicine, American University of Beirut, P.O. Box 11-0236, Beirut, Lebanon; had29@mail.aub.edu; 4 Department of Anatomy, Cell Biology and Physiological Sciences, Faculty of Medicine, American University of Beirut, P.O. Box 11-0236, Beirut, Lebanon; ae49@aub.edu.lb; 5 Department of Chemistry, Faculty of Arts and Sciences, American University of Beirut, P.O. Box 11-0236, Beirut, Lebanon; tg02@aub.edu.lb; 6 Department of Medicinal Chemistry and Pharmacognosy, Faculty of Pharmacy, Jordan University of Science and Technology, P.O. Box 3030, Irbid 22110, Jordan; telimat@just.edu.jo; 7 College of Pharmacy, Qatar University, Doha P.O. Box 2713, Qatar; feras.alali@qu.edu.qa; 8 Department of Nutrition, University of Petra, Amman, P.O. Box 961343, Amman 11196, Jordan; abadran@uop.edu.jo; 9 Department of Biomedical Sciences, College of Health Sciences, Qatar University, Doha P.O. Box 2713, Qatar

**Keywords:** vascular smooth muscle cells, inflammation, phenotypic switch, homoisoflavonoids, 7-*O*-methylpunctatin, arterioles

## Abstract

Remodeling of arterioles is a pivotal event in the manifestation of many inflammation-based cardio-vasculopathologies, such as hypertension. During these remodeling events, vascular smooth muscle cells (VSMCs) switch from a contractile to a synthetic phenotype. The latter is characterized by increased proliferation, migration, and invasion. Compounds with anti-inflammatory actions have been successful in attenuating this phenotypic switch. While the vast majority of studies investigating phenotypic modulation were undertaken in VSMCs isolated from large vessels, little is known about the effect of such compounds on phenotypic switch in VSMCs of microvessels (microVSMCs). We have recently characterized a novel homoisoflavonoid that we called 7-*O*-methylpunctatin (MP). In this study, we show that MP decreased FBS-induced cell proliferation, migration, invasion, and adhesion. MP also attenuated adhesion of THP-1 monocytes to microVSMCs, abolished FBS-induced expression of MMP-2, MMP-9, and NF-κB, as well as reduced activation of ERK1/2 and FAK. Furthermore, MP-treated VSMCs showed an increase in early (myocardin, SM-22α, SM-α) and mid-term (calponin and caldesmon) differentiation markers and a decrease in osteopontin, a protein highly expressed in synthetic VSMCs. MP also reduced transcription of cyclin D1, CDK4 but increased protein levels of p21 and p27. Taken together, these results corroborate an anti-inflammatory action of MP on human microVSMCs. Therefore, by inhibiting the synthetic phenotype of microVSMCs, MP may be a promising modulator for inflammation-induced arteriolar pathophysiology.

## 1. Introduction

Arterioles are internally wrapped with one or more layers of vascular smooth muscle cells (VSMCs) [1]. Under normal physiological conditions, VSMCs assume a contractile phenotype [2]. It is this phenotype that is largely responsible for the contractility of blood vessels, allowing them to tightly control vasotone and regulate flow both under physiologic and pathophysiologic conditions [2,3,4]. In response to inflammatory cues, VSMCs switch from the contractile to a dedifferentiated synthetic phenotype, with increased proliferative and migratory abilities [2]. This VSMC phenotypic switch plays a key role in vascular/arteriolar inflammation and remodeling [5].

The use of herbal medicine in the prevention and treatment of cardiovascular disease (CVD) has been substantially growing [6,7]. Remarkably, the PubMed database contains more than 600 clinical trials and around 3300 publications dealing with herbal drugs for CVDs [7]. These reports provide scientific evidence on the beneficial role of herbal medicine in CVD. Among the many herbal compounds associated with cardio-vasculoprotective effects are flavonoids [8,9,10,11,12]. For instance, the flavonoid-rich herb *Scutellaria baicalensis* was reported to confer protection against ischemic heart disease [13]. Likewise, flavonoids are known to ameliorate atherosclerosis [14] and exert an antihypertensive effect [15]. Importantly, several studies tested the effect of many chemicals/flavonoids with anti-inflammatory actions on vascular cells. Nonetheless, most of these drugs were tested on VSMCs isolated from large vessels [16,17,18,19]. Indeed, very little is known about compounds targeting arteriolar inflammation.

A special class of flavonoids distinguished by one additional carbon atom on their carbon cytoskeleton are the homoisoflavonoids (3-benzylidenechroman-4-ones) [20]. They constitute a rare class of natural compounds [20]. More than 240 natural homoisoflavonoids have so far been reported, all restricted to only six plant families: Fabaceae, Asparagaceae, Polygonaceae, Portulacaceae, Orchidaceae, and Gentianaceae [16,20,21]. Recently, homoisoflavonoids have been receiving increased interest due to their broad spectrum of biological effects [20]. These include anti-inflammatory [22], anti-hyperglycemic [23], anti-mutagenic [24], anti-microbial [25], antiviral [26], and anti-oxidant activities [27]. The anti-oxidant effect seems to be the most important and most extensively studied owing to its potentially beneficial effects in diabetes and inflammation [28] and CVD [29]. For instance, *Ophiopogonin japonicus*, rich in anti-oxidative homoisoflavonoids, appears to be effective in treating myocardial ischemia and arrhythmias [29]. Thus, by virtue of their anti-oxidative potential, homoisoflavonoid-rich plants may be regarded as an important resource in the management or treatment of CVD.

**Figure 1:**
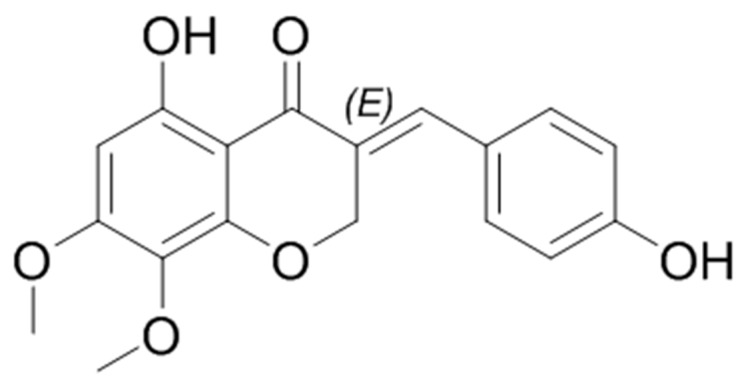
The chemical structure of 7-*O*-methylpunctatin (MP).

*Bellevalia eigii* Feinbrun is a perennial plant belonging to the family Asparagaceae [16,30]. It is native to Mediterranean region and Sinai [31] and is widespread in Jordan, where it is known among local people as “the Jordan Valley onion” [16]. From the bulbs of *Bellevalia eigii* Feinbrun, we recently isolated, purified and characterized a new compound, 7-*O*-methylpunctatin (MP) [16] (Figure 1). Here, we sought to determine the effect MP on fetal bovine serum (FBS)-induced inflammation of human VSMCs extracted from dermal arterioles.

## 2. Materials and Methods

### 2.1. Reagents

Anti-Calponin antibody (ab46794), anti-Caldesmon antibody (ab32330), anti-β actin antibody (ab119716), anti-ERK1/2 antibody (ab17942), anti-ERK1 (phospho Y204) + ERK2 (phospho Y187) antibody (ab47339), anti-FAK antibody (ab61113), anti-FAK antibody (phospho Y397, ab81298), HRP-conjugated Goat Anti-Mouse antibody (ab97040), HRP-conjugated Goat Anti-Rabbit antibody (ab ab150080), and Tetrazolium (ab146345) were purchased from Abcam (Cambridge, UK). Anti-GAPDH antibody (2118), anti-caspase-3 (8G10), anti- nuclear factor (NF)-κB p65 antibody (3034), anti-IκB antibody (9242), and anti-IκBα (phospho Ser32/36, 9246) were obtained from Cell Signaling Technology (Leiden, The Netherlands). Anti-p21antibody (sc-397) and anti-p27 antibody (sc-1641) were purchased from Santa Cruz Biotechnology (Dallas, TX, USA). Dulbecco’s Modified Eagle’s Medium/Nutrient Mixture F-12 Ham DMEM: F12 (BE12-719F), RPMI-1640, Penicillin/Streptomycin (17-602E), and Trypsin (BE02-007E), DMSO (0231) were obtained from Lonza (Basel, Switzerland). Fetal Bovine Serum FBS (F9665), Phosphorous Buffer Saline PBS (D1408), and Propidium Iodide (P4170) were purchased from Sigma-Aldrich (Schnelldorf, Germany). MMP-2 and MMP-9 ELISA kits were obtained from R&D Systems (Minneapolis, MN, USA), *DC* Protein Assay kit and ClarityWestern ECL Substrate from Bio-rad (Irvine, CA, USA), BrdU kit from Roche Diagnostics (Penzberg, Germany), Luciferase Assay Kit from Promega (Fitchburg, WI, USA), Moloney murine leukemia virus reverse transcriptase (RT) from Invitrogen (Carlsbad, CA, USA), and SYBR Green fluorophore from SuperArray Bioscience Corporation (Frederick, MD, USA).

### 2.2. Cell Culture

Human arteriolar smooth muscle cells were extracted by the non-enzymatic sprouting method from post-circumcision tissue of a newborn boy. No IRB approval is needed as this source is considered clinical waste. Cells were grown in Ham’s Growth medium (DMEM: F12, 50:50; supplemented with 10% FBS, and 1% penicillin/streptomycin). Only cells of passages 8–11 were used. Before treatment, cells were synchronized by starvation in a quiescent serum-free medium (DMEM: F12, 50:50, 0.5% FBS, 1% penicillin/streptomycin) for 48 h, as previously described [32]. THP-1 cells were cultured in RPMI-1640 and supplemented with 10% FBS and 1% penicillin/streptomycin. Cells were maintained in a humidified incubator at 37 °C with 5% CO_2_ atmosphere.

### 2.3. Preparation of 7-O-methylpunctatin 

Extraction, characterization, and purification of MP was done as we recently reported [16]. MP was stored at −20 °C, and for cell treatment, it was dissolved in DMSO. The dissolved compound was stored in the dark at −20 °C.

### 2.4. MTT Assay

VSMCs were grown in 96-well plate until they reached 30–40% confluence. Then cells were starved in serum-free medium for 48 hrs. Following starvation, cells were treated with increasing concentrations of MP for 24, 48, and 72 h. MTT solution (20 µL, 5 mg/mL) was added to each well, and cells were incubated for an hour in a 5% CO_2_ incubator. The medium was then removed, and 200 µL DMSO was added to each well. The plate was placed on a shaker for 15 min to allow for the dissolution of formazan crystals. Using an ELISA Multiscan EX Reader (Thermo Fisher, Vantaa, Finland), optical density was read at 550 nm. Absorbance is directly proportional to cell viability.

### 2.5. BrdU Incorporation Assay

Here, five thousand cells/well were seeded into 96-well plates. Cells were then starved for 48 h before commencing any treatment. Cell proliferation was then measured with BrdU kit (Roche Diagnostics, Penzberg, Germany) following the manufacturer’s protocol. Optical density was measured using a microplate reader spectrophotometer at excitation wavelength 450 nm.

### 2.6. Cell Cycle Analysis

Cells were made quiescent by culturing in starvation medium for 48 h. After starvation, cells were treated for 48 h with complete medium in the absence or presence of MP. They were then washed with PBS, trypsinized, and collected by centrifugation. After washing twice with ice-cold PBS, cells were re-suspended in 500 µL PBS. For permeabilization and fixation, 2 mL of ice-cold pure ethanol was added for 15 min. The cell suspension was centrifuged, and the cell pellet was washed twice with PBS. Cells were then incubated for 10 min in 1 mg/mL of propidium iodide in PBS. Propidium iodide (PI) fluorescence was read using Guava EasyCyte8 Flow Cytometer (Luminex, Hayward, CA, USA). Cell cycle analysis was done using Guava Soft 2.7 software.

### 2.7. RT-PCR

Cells were seeded and allowed to grow in complete medium, then starved for 48 h. Total RNA was extracted using Nucleospin RNA II kit as per the manufacturer’s protocols (Machery Nagel, Germany). cDNA was then synthesized using 1 µg of total RNA by RevertAid 1st strand cDNA synthesis kit (Thermo Fisher ScientificF). RT-PCR was then performed using the iQ SYBR green supermix. Using serial dilutions of cDNA of positive controls for each gene of interest, standard curves are determined and plotted, and then the threshold cycle value (Ct) obtained for each gene and normalized to the housekeeping gene GAPDH (internal control). The ΔΔCt method was used to analyze expression changes between the different conditions, where the control untreated group value is set to one. Gene sequences were amplified using the following primers:

- Cyclin D1F: TCCTGTGCTGCGAAGTGGAAAC;
- Cyclin D1R: AAATCGTGCGGGGTCATTGC;
- cdk4F: AAGAGTGTGAGAGTCCCCAATGG;
- cdk4R: GATTTTGCCCAACTGGTCGG;
- Myocardin F: GAGAGGTCCATTCCAACTGC;
- Myocardin R: GGGCTGTGAGGCTGAGTC;
- SM-22α F: TCCAGGTCTGGCTGAAGAATGG;
- SM-22α R: CTGCTCCATCTGCTTGAAGACC;
- SM-α F: ACTGAGCGTGGCTATTCCTCCGTT
- SM-α R: GCAGTGGCCATCTCATTTTCA;
- GAPDH F: CGCTCTCTGCTCCTCCTGTTC;
- GAPDH R: TTGACTCCGACCTTCACCTTCC.

### 2.8. Phase Contrast Microscopy

VSMCs were grown in 6-well plates. Cells were then starved for 48 h then treated with complete medium in the absence or presence of MP. Images were acquired using a phase-contrast microscope (Zeiss, Oberkochen, Germany) after 24, 48, and 72 h.

### 2.9. Scanning Electron Microscopy 

Cells were cultured in complete medium on coverslips in 12-well plates. At 80% confluency, cells were starved for 48 h, then treated with complete medium in the absence or presence MP for 48 hrs. Wells were then washed and cells fixed with 2.5% glutaraldehyde for 45 min at 4 °C. After washing with PBS, cells were dehydrated with increasing ethanol concentrations (25, 50, 75, 95, and 100%) for 5 min per incubation. The coverslips were mounted on scanning electronic miscoscope (SEM) stub, cells were sputtered with gold and images were acquired using Tescan SEM (MIRA3 software; Brno; Czech Republic).

### 2.10. Wound Healing (Scratch) assay 

Cells were cultured in 12-well plates until 90–95% confluent. They were then incubated in quiescent medium (0.5% FBS) for 48 h. Using a 10 μL sterile pipette tip, a scratch was made on the cellular monolayer. Wells were washed with PBS to remove cellular debris, and medium was replenished in the absence or presence of MP. Wound healing was monitored at 0, 2, 4, 6, 8, 12, and 24 h, and photomicrographs were taken using a Zeiss phase contrast microscope. ZEN imaging software (blue edition) from Zeiss was used to measure the width of the scratch.

### 2.11. Invasion Assay

Transwell inserts were coated with matrigel and allowed to dry overnight under ultraviolet light. Cells in serum-free media were seeded onto the rehydrated upper transwell chamber in the absence or presence of MP. The lower chamber was loaded with complete medium, acting as a chemotactic attractant. Cells were then incubated at 37 °C for 24 h. After treatment, the medium was aspired, and wells were washed with PBS. Non-invading cells were removed from the upper surface with a cotton swab, whereas invading cells were fixed with methanol and stained with DAPI. The membrane was cut with a blade and mounted on an anti-fade agent. Slides were observed under Zeiss Axio fluorescent microscope. Cells from at least five different fields were counted.

### 2.12. Cell Adhesion Assay

Cells in starvation medium were seeded in 6-well plates and allowed to adhere for 1 h at 37 °C. Then, wells were gently washed with PBS to remove non-adherent cells. Images were acquired using Zeiss phase contrast microscope and adherent cells were counted.

### 2.13. Monocyte Adhesion Assay

Cells were grown in complete medium until confluence. Cells were then treated with MP for 1hour followed by treatment with phorbol myristate acetate (PMA) for 20 h. THP-1 cells labelled with NucBlue (Thermo Fischer Scientific) were added over the VSMC monolayer and allowed to adhere for 30 min. Non-adherent THP-1 cells were removed by washing the wells with PBS. Images of the adherent THP-1 cells were acquired using Zeiss Axio fluorescent microscope. In addition, quantitative analysis was done by measuring the fluorescence intensity of five random fields of photomicrographs.

### 2.14. Measurement of MMP-2 and MMP-9

Cells were grown to a subconfluent level and then starved for 48 h. Following treatment with the respective conditions, medium of each condition was collected for MMP-2 and MMP-9 detection using ELISA kits (R&D Systems), as recommended by the manufacturer and we recently reported [33].

### 2.15. Actin Staining

At 30–40% confluence, cells were starved in quiescent medium for 48 h. Following treatment, cells were washed twice with PBS, fixed with 4% paraformaldehyde, and permeabilized with 0.1% Triton X-100. Cells were then washed again and incubated with Rhodamine phalloidin stain for 1 h in the dark. Cells were washed again, and nuclei were counter-stained with DAPI for 15 min at room temperature. Cells were mounted on an anti-fading agent and visualized using Zeiss Axio fluorescent microscope.

### 2.16. Luciferase Reporter Assay

As we previously reported [34], cells were transiently transfected with NF-κB-driven promoter luciferase using Amaxa Nucleofector (Amaxa Biosystems, Gaithersburg, MD, USA) according to the manufacturer’s protocol. Renilla luciferase vector was used as an internal control, to which firefly luciferase values were normalized. Following transfection, cells were allowed to recover overnight, and then starved for 48 h. After treatment, cells were washed and lysed in luciferase lysis buffer (Promega), and luciferase activity was measured.

### 2.17. Western Blotting

Cells were washed with PBS and then lysed using 2% SDS, 60 mM Tris lysis buffer (pH 6.8) as previously described [35]. Proteins were quantified using *DC* Protein Assay and equal amounts of protein (20–30 µg) were loaded and separated using 5–11% SDS-PAGE. Proteins were then transferred onto polyvinylidene difluoride (PVDF) membrane (Biorad). After blocking in 5% fat-free milk in TBS-T, 1 h at room temperature, the membrane was incubated overnight with the relevant primary antibody at 4 °C. The membrane was then washed thrice with TBS-T for 10 min each and incubated with the appropriate HRP-conjugated secondary antibody for 1 h at room temperature. The membrane was washed again (three times with TBS-T, 10 min each) and then developed using enhanced chemiluminescence (ECL clarity, Biorad) and quantified using Chemidoc MP Imaging system (Biorad).

### 2.18. Statistical Analysis

Statistical analyses were performed by student’s *t*-test for either paired or unpaired observations. For multiple comparisons, ANOVA was used—either one-way ANOVA (with Dunnett’s post hoc test) or two-way ANOVA (with Tukey-Kramer’s post hoc test). Except for Western blotting, experiments were performed at least three times, and each time was made of triplicate wells. The average of the triplicate from each experiment (individual mean) was calculated, and these means were then averaged. Data were presented as mean ± standard error of the mean (SEM). A *p*-value of less than 0.05 was considered as significant.

## 3. Results

### 3.1. MP Inhibits FBS-Induced VSMC Proliferation

**Figure 2:**
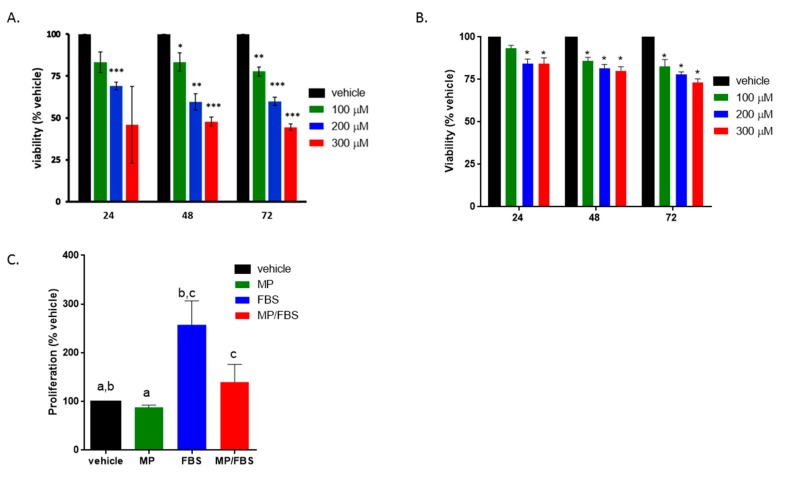
MP attenuates basal and (fetal bovine serum) FBS-induced vascular smooth muscle cells (VSMC) proliferation. Cells were treated with increasing concentrations of MP (100, 200, and 300 µM) for 24, 48, and 72 h in the presence (**A**) or absence (**B**) of FBS. Cell viability was assessed by the overall metabolic activity measured by MTT. *p < 0.05, **p < 0.01 and ***p < 0.005. Two-way ANOVA was performed. (**C**) Cells were grown in starvation or complete medium, in presence or absence of MP (100 μM). DNA synthesis was assessed BrdU incorporation assay. Values are calculated as % of the corresponding vehicle control value and represented as mean ± standard error of the mean (SEM) of three different experiments, each run in triplicate. Bars with same letters are statistically significant. One-way ANOVA followed by Tukey’s test was performed.

Cells were stimulated with FBS then were treated with or without MP. MP inhibited proliferation in a concentration- and time-dependent manner (Figure 2A). At the concentrations of 200 and 300 µM, MP significantly reduced the number of viable cells at 24, 48, and 72 h. However, the lowest MP concentration (100 µM) caused a significant reduction only at 48 and 72 h (100 vs 83% and 100 vs 77% respectively; *p* < 0.05 for both). A similar result was observed when FBS-unchallenged (i.e., quiescent) cells were treated with increasing concentrations of MP (100, 200, and 300 µM) (Figure 2B). This suggests that MP inhibits basal and FBS-induced proliferation of these cells.

In order to determine whether the anti-proliferative effect of MP is associated with a change in DNA synthesis, BrdU incorporation assay was employed. MP slightly but significantly decreased basal BrdU incorporation (100 vs 88%; *p* < 0.05) (Figure 2C). As expected, FBS induced a significant increase in DNA synthesis (100 vs 258 ± 28%; *p* < 0.05) (Figure 2C). This increase was potently diminished by MP (258 ± 28% vs 140 ± 20%; *p* < 0.01). These results are in line with the anti-proliferative effect of MP evaluated by MTT.

### 3.2. MP Induces Cell Cycle Arrest of FBS-Induced VSMC

**Figure 3:**
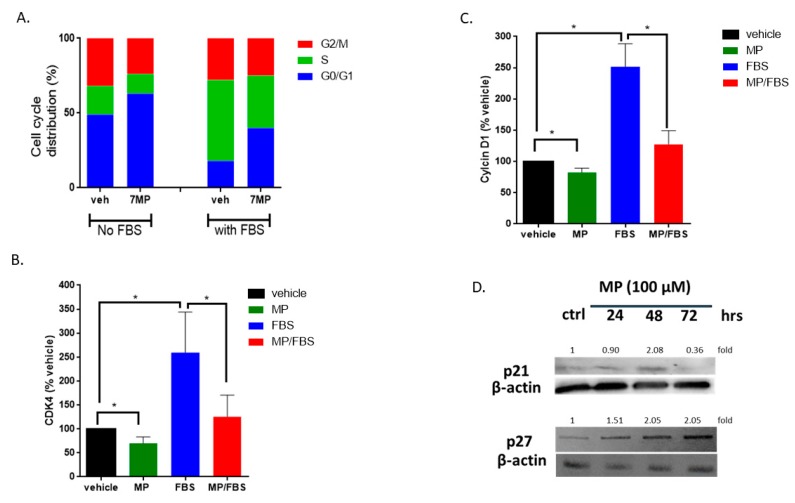
MP blocks VSMCs in G_0_/G_1_ phase of cell cycle. (**A**) Cells were treated with MP (100 μM) for 48 h, stained with PI, then sorted by flow cytometer. Data represent the mean of three independent experiments. (**B**) and (**C**) Cells were grown in starvation or complete medium, in presence or absence of MP (100 µM). The expression levels of CDK4 (**B**) and Cyclin D1 (**C**) were determined by RT-PCR. Values are calculated as % of the corresponding vehicle control value and represented as mean ± SEM of three replicates. (*p < 0.05). (**D**) Cells were treated with MP (100 µM) for 24, 48, and 72 h. The expression of p21 and p27 was detected by Western blotting. Values mean fold change of three replicates. One-way ANOVA followed by Tukey’s test was performed for all panels.

Having established that MP attenuates FBS-induced cell proliferation, we next sought to assess the effect of MP on cell cycle progression. As shown in Figure 3A, MP increased the G_0_/G_1_ cell population, while decreasing those in the S and G_2_/M phases. Expectedly, treatment with FBS decreased the percentage of cells in G_0_/G and increased the percentage of cells in the S phase (Figure 3A). These FBS-induced changes in the cell cycle profile were attenuated by pretreatment with MP. This indicates that MP inhibited cell proliferation by arresting the cells in the G_0_/G_1_ phase.

### 3.3. MP Downregulates the Expression of Cyclin D1 and CDK4 and Upregulates the Expression of CDK Inhibitors, p21 and p27, in VSMCs

To further validate our results, we sought to characterize the changes in the expression of factors directly involved in cell cycle regulation. Here, we looked at the expression level of cyclin D1, CDK4, p21 and p27. RT-PCR analysis showed that MP significantly decreased the mRNA level of cyclin D1 and CDK4 (Figure 3B,C). As expected, treatment with FBS induced an increase in the transcript level of both cyclin D1 (100 vs 252 ± 21%; *p* < 0.05) and CDK4 (100 vs 259 ± 49%; *p* < 0.05) (Figure 3B,C). This increase was abrogated by MP (252 ± 21% vs 127 ± 12% for cyclin D1 or 259 ± 49% vs 125 ± 26% for CDK4) (Figure 3B,C). Furthermore, MP induced an increase in the expression of p21 and p27 (Figure 3D). While the upregulation of p21 was noticeable at 24 and 48 h (Figure 3D), p27 was upregulated only after 48 h of treatment with MP (Figure 3D). These results further reinforce our hypothesis that MP attenuates FBS-induced cell cycle progression by inhibiting the escape from G_1_ phase.

### 3.4. MP Induces VSMC Apoptosis

**Figure 4:**
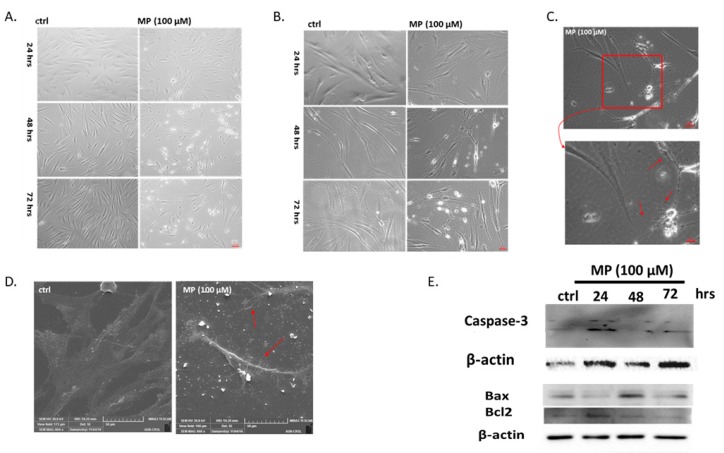
MP induces VSMC apoptosis. Cells were treated with MP (100 μM) for 24, 48, and 72 h. Micrographs were captured at magnifications of (**A**) 4×, (**B**) 10×, and (**C)** 20×. (**C**) The area in the red square was captured at 100× magnification (lower micrograph). (**D**): Representative SEM images of MP-treated cells. Scale bars, 50 μm. In (**C**) and (**D**): Red arrows point at cytoplasmic extensions. (**E**) Cells were treated with MP (100 μM) for 24, 48, and 72 h. Activation of caspase-3 and the expression levels of Bax and Bcl2 were detected by Western blotting. Values mean fold change of three independent experiments. One-way ANOVA followed by Tukey’s test was performed.

It is well-established that cellular morphologic changes greatly reflect as well as affect cellular function [36,37,38]. For instance, cells undergoing apoptosis show distinguished morphological features such as cell shrinkage and cytoplasmic extensions [39]. Light microscopic examination of MP-treated cells revealed morphological changes indicative of a loss in the VSMC characteristic spindle shape. These changes occurred 48 and 72 h post treatment (Figure 4A). Indeed, cells adopted a round translucent morphology (Figure 4A), likely indicative of cell death. Higher magnification showed shrunken and smaller-sized cells (Figure 4B). Moreover, treated cells exhibited long string-like cytoplasmic extensions with blebs attached to their ends (Figure 4C; red arrows). All these changes are characteristic of apoptotic cells [40,41]. Using SEM, the cellular ultrastructure revealed the presence of cytoplasmic protrusions known as apoptopodia (Figure 4D; red arrows). Collectively, these observations indicate that MP induces apoptosis of microvascular smooth muscle cells.

To validate our finding, we determined the effect of MP on caspase-3 cleavage and Bax/Bcl2 ratio. We found that caspase-3 was not activated in MP-treated VSMC for 24 h (Figure 4E). This result is in agreement with cell viability results and cell morphology images. However, longer exposure (48 and 72 h) to MP activated apoptotic cell death revealed by the cleavage of caspase 3 (Figure 4E). In addition, MP induced an increase in the Bax/Bcl2 ratio after 48 or 72 h of treatment (Figure 4E), suggesting the activation of the intrinsic apoptotic pathway.

### 3.5. MP Attenuates FBS-Induced VSMC Migration, Invasion, and Adhesion 

**Figure 5:**
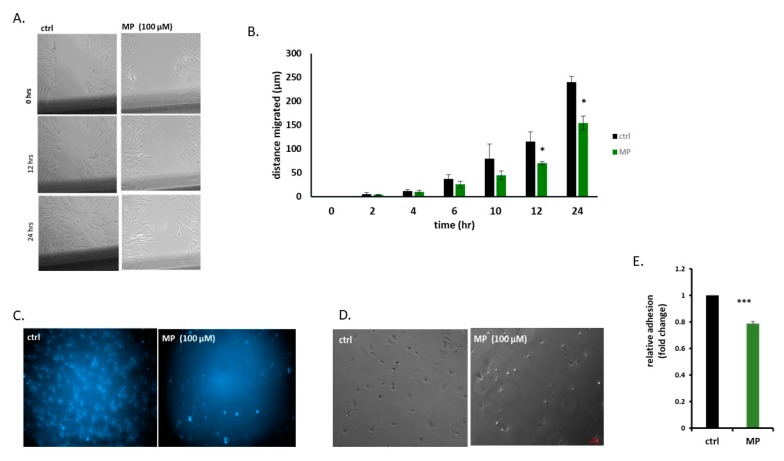
MP inhibits VSMC migration, invasion, and adhesion. (**A**) Cells were treated with MP (100 µM) and cell migration was assessed by scratch assay. Images were taken at the indicated time points (Scale bar, 50 µm). (**B**) Values are represented as mean ± SEM of distance migrated (*n* = 3 replicates) (*p < 0.05). (**C**) Cells were treated with MP (100 µM). Cell invasion was evaluated using invasion assay. Representative photomicrographs showing the effect of MP on invading cells. (**D**) Cells were treated with MP (100 µM) and allowed to adhere for 1 hr. Representative photomicrographs of the effect of MP on VSMC adhesion. Scale bar, 50 µm. (**E**) Values are represented as mean ± SEM of relative fold inhibition of vehicle-treated cells (*p < 0.05). One-way ANOVA was performed for all panels.

The effect of MP on FBS-induced migration was examined using scratch assay. MP, at a concentration of 100 µM, significantly attenuated wound-healing (*p* < 0.05) after 12 h (Figure 5A,B). At this time point, no cytotoxic effect of MP was observed (data not shown) (Figure 5A,B), indicating that the anti-migratory capacity is independent of MP’s anti-proliferative effect. In addition, we evaluated the effect of MP on the invasive capacity of VSMCs using Matrigel-coated Boyden chambers. Our results showed that MP inhibited FBS-induced invasiveness (Figure 5C).

Because cell adhesion to its substratum is critical for cell migration and invasion, we next determined the effect of MP on VSMC adhesion. We found that MP significantly inhibited VSMC adhesion as shown in Figure 5D,E.

### 3.6. MP Inhibits MMP-2 and MMP-9 Secretion in VSMCs

**Figure 6:**
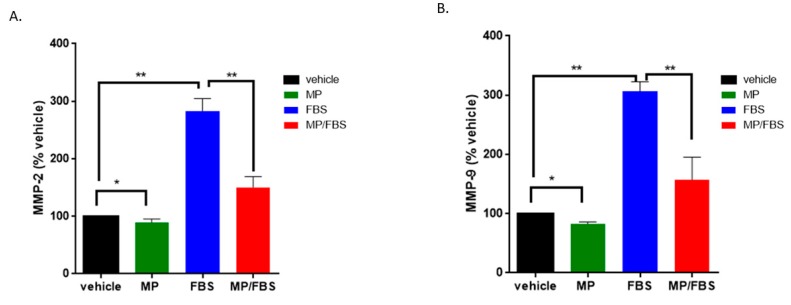
MP inhibits basal and FBS-induced MMP-2 and MMP-9 secretion. Cells were grown in starvation or complete medium, in presence or absence MP (100 μM). Levels of secreted MMP-2 (**A**) and MMP-9 (**B**) were evaluated by ELISA. Data represented are mean ± SEM of % MMP level in the corresponding vehicle-treated well. (*p < 0.05, **p < 0.01). One-way ANOVA followed by Tukey’s test was performed.

Matrix metalloproteases-2 and -9 (MMP-2 and MMP-9) are known to play a major role in vascular remodeling. Specifically, the activation of MMP-2 and MMP-9 in response to inflammatory stimuli leads to ECM degradation, thus facilitating VSMC migration and invasion [42,43,44]. Here, our results show that MP significantly decreased the levels of secreted MMP-2 and MMP-9. Specifically, MP slightly but significantly reduced basal levels of secreted MMP-2 (100 vs 89 ± 3%; *p* < 0.05) (Figure 6A) and MMP-9 (100 vs 83 ± 2%; *p* < 0.05) (Figure 6B). Stimulation with FBS induced a profound increase in MMP-2 (100 vs 283 ± 12%; *p* < 0.01) and MMP-9 (100 vs 307 ± 9%; *p* < 0.01) (Figure 6A,B). This increase was significantly attenuated by pretreatment with MP (283 ± 12% vs 150 ± 11% or 307 ± 9% vs 157 ± 22%, for MMP-2 and MMP-9 respectively; *p* < 0.01 for both) (Figure 6A,B).

### 3.7. MP Decreases the Phosphorylation of ERK1/2 and FAK

**Figure 7:**
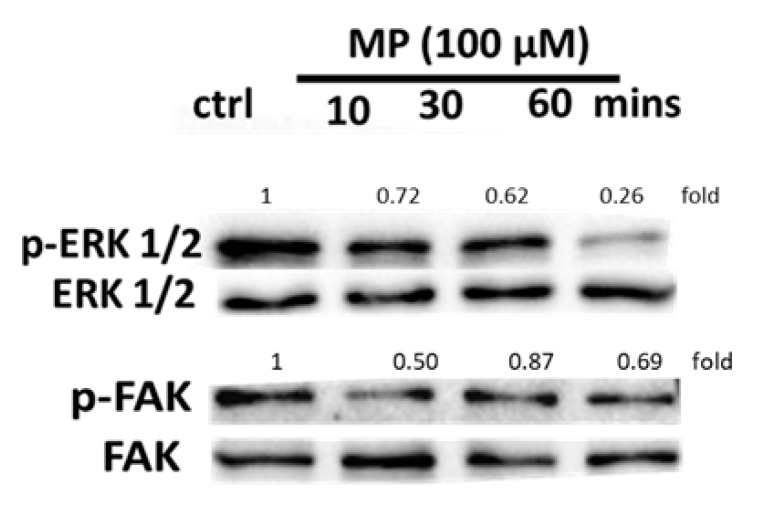
MP attenuates ERK1/2 and FAK phosphorylation in a time-dependent manner. Cells were treated with MP (100 μM) for 10, 30, and 60 min. The phosphorylation levels of ERK1/2 and FAK were determined by Western blotting. Values mean fold change of three independent experiments.

Activation of the ERK1/2 pathway plays a key role in VSMC proliferation and migration [45,46,47]. In addition, FAK activation is associated with cell migration and adhesion [48]. Thus, we investigated the effect of MP on the phosphorylation of ERK and FAK using Western blotting. We found that MP induced a decrease in ERK1/2 phosphorylation in a time-dependent manner (Figure 7). Moreover, FAK phosphorylation decreased as early as 10 min post-MP treatment (Figure 7).

### 3.8. MP Increases the Expression of Early and Mid-Term Differentiation Markers and Decreases the Expression of a De-differentiation Marker

VSMC phenotype, whether contractile or synthetic, may be defined by the level of expression of specific markers. The contractile phenotype is characterized by differentiation markers that are grouped into early, mid-term, and late differentiation markers [49]. On the other hand, synthetic VSMCs secrete many ECM proteins, including osteopontin and osteonectin [50,51].

**Figure 8:**
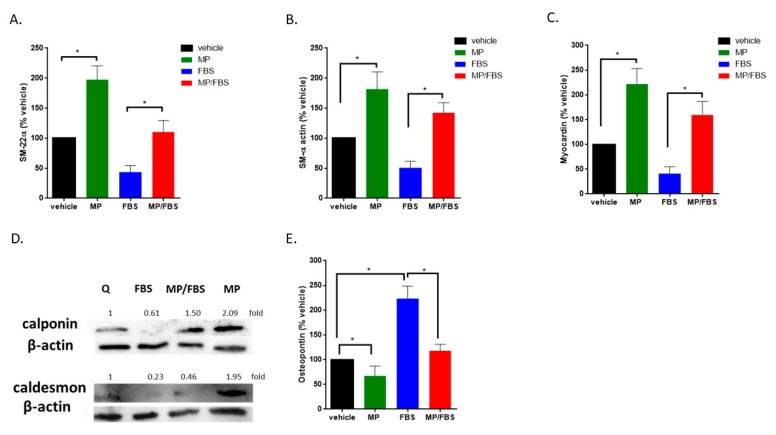
MP increases the expression of basal and FBS-attenuated differentiation markers and decreases basal and FBS-induced expression of osteopontin. Cells were grown in starvation or complete medium, in presence or absence MP (100 μM). Expression levels of contractile differentiation markers (**A**) SM-22α, (**B**) SM-α, (**C**) myocardin were evaluated by RT-PCR. Values represented are mean ± SEM of % vehicle control. (*p < 0.05). (**D**) Cells were grown in starvation or complete medium, in presence or absence MP (100 μM). The expression of differentiation markers, calponin and caldesmon, was determined by Western blotting. Values mean fold change of three independent experiments. (**E**) Cells were grown in starvation or complete medium, in presence or absence MP (100 μM). The expression level of osteopontin was asssessed by RT-PCR. Values represented are mean ± SEM of % vehicle control. (*p < 0.05). One-way ANOVA followed by Tukey’s test was performed.

Here, we show that MP significantly increased the expression of the early differentiation markers, SM22-α, SM α-actin, and myocardin (100 vs 197 ± 16%; 100 vs 181 ± 20%; or 100 vs 222 ± 22%; for SM22-α, SM α-actin, and myocardin respectively; *p* < 0.01 for all) in quiescent cells (Figure 8A–C). As expected, treatment with FBS induced a decrease in the expression of these markers. This decrease was greatly attenuated by MP (FBS alone versus FBS plus MP: 43 ± 8% vs 110 ± 14%; 51 ± 8% vs 142 ± 12%; or 41 ± 10% vs 160 ± 19%; for SM22-α, SM α-actin, or myocardin respectively; *p* < 0.01 for all). Similar results were obtained for mid-term differentiation markers, calponin and caldesmon (Figure 8D) Moreover, MP abrogated the basal (100 vs 67 ± 14%; *p* < 0.01) and FBS-induced (223 ± 18% vs 118 ± 9%; *p* < 0.01) expression of osteopontin, a glycoprotein secreted by synthetic VSMCs (Figure 8E). These results indicate that MP drives VSMCs towards a contractile phenotype via increasing the expression of differentiation markers and decreasing the expression of osteopontin in quiescent and FBS-induced cells.

### 3.9. MP Inhibits Actin Polymerization

**Figure 9:**
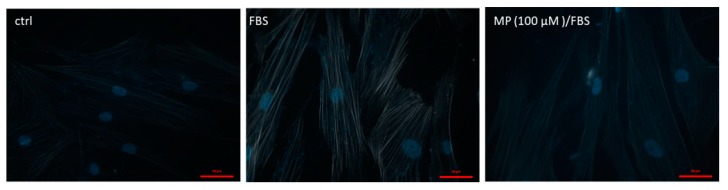
MP attenuates FBS-induced actin polymerization. Cells were treated with FBS for 24 h in the presence or absence of MP (100 μM). Cells were then stained with phalloidin and actin polymerization was assessed. Representative micrographs showing the effect of MP on FBS-induced actin-polymerization. Scale bar, 50 µm.

The actin cytoskeleton in VSMCs is dynamic and responds to external stimuli by polymerization of globular (G) actin to filamentous (F) actin [52]. To test the effect of MP on FBS-induced actin polymerization, phalloidin stain was employed. Phalloidin binds to F-actin and prevents their depolymerization. As expected, FBS treatment induced actin polymerization. This FBS-induced polymerization was inhibited by MP pretreatment as shown in Figure 9.

### 3.10. MP Inhibits PMA-induced Adhesion of THP-1 Monocytes on VSMCs

**Figure 10:**
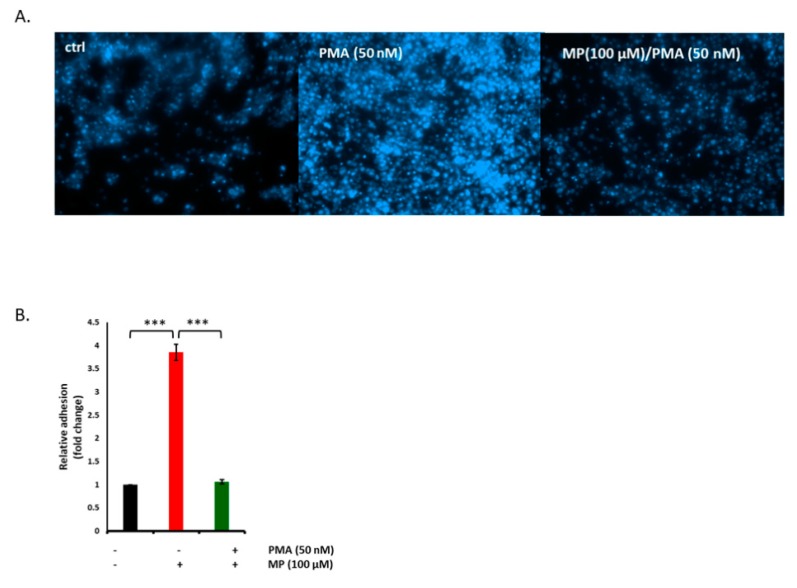
MP abolishes phorbol myristate acetate (PMA)-evoked adhesion THP-1 cells to VSMCs. Cells were treated with PMA (50 nM) in presence or absence of MP (100 µM), and adhesion of THP-1 cells was examined by monocyte adhesion assay (**A**). Representative photomicrographs of the effect of MP on THP-1 adhesion to VSMC. (**B**) Quantitation of mean fluorescence intensity of adherent THP-1, assessed at 5 different regions; *** *p* < 0.001. Values are represented as mean ± SEM of relative fold adhesion of vehicle-treated cells. One-way ANOVA followed by Tukey’s test was performed.

Monocyte adhesion to VSMC takes place in many vasculopathies including atherosclerosis, thrombosis, and restenosis [53]. We sought to determine the effect on MP on monocyte adhesion to PMA-induced VSMCs. Toward this, THP-1 cells were incubated with PMA-stimulated VSMCs, with or without pretreatment with MP. In the absence of PMA, the number of THP-1 cells adhered to VSMCs was expectedly minimal. However, stimulation of VSMCs with PMA for 20 h lead to significant increase in the number of adherent THP-1 cells (Figure 10A). Indeed a 3-fold increase in monocyte adhesion was observed under these conditions (Figure 10B). Interestingly, this increase was completely abolished when VSMC were pretreated with 100 µM of MP (Figure 10A,B).

### 3.11. MP Inhibits FBS-Induced Expression of NF-κB in a Concentration-Dependent Manner

**Figure 11:**
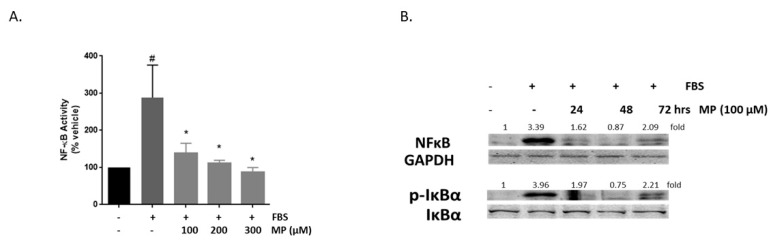
MP inhibits FBS-induced NF-κB expression in a time- and concentration-dependent manner, and attenuates the activation of its inhibitor, IκBα. (**A**) Cells were treated with FBS in the absence or presence of increasing concentrations of MP (100, 200, and 300 µM). NF-κB expression was determined by luciferase assay. Values represented are mean ± SEM. # denotes *p* < 0.05 (FBS versus vehicle) and * denotes *p* < 0.05 (MP + FBS vs FBS)). (**B**) Cells were treated with FBS in the presence or absence of MP (100 μM) for 24, 48, and 72 h. Expression of NF-κB and phosphorylation of IKBα were detected by Western blotting. Values mean fold change of three independent experiments. One-way ANOVA followed by Tukey’s test was performed.

NF-κB transcription factor is a key regulator of vascular inflammatory responses [54]. Here, the effect of MP on the expression of NF-κB and the phosphorylation of its inhibitor protein, IκB, were evaluated. As shown in Figure 11A, FBS evoked a significant increase in NF-κB transcription (100 vs 288 ± 62%; *p* < 0.05). This increase was attenuated by MP in a concentration-dependent manner (288 ± 62% vs 138 ± 19%, 111 ± 6% or 87 ± 9% for 100, 200, and 300 µM respectively; *p* < 0.01 for all). Western blotting analysis showed that whereas FBS induced an increase in NF-κB expression, pretreatment with MP abolished its activation in a time-dependent manner (Figure 11B). Furthermore, pretreatment with MP inhibited FBS-induced phosphorylation of IκBα (Figure 11B).

## 4. Discussion

Inflammation of arterioles has recently emerged as a key event in the manifestation of many diseases [1,55,56]. These include diabetes and inflammation-based disorders such as chronic obstructive pulmonary disease, inflammatory bowel diseases, cystic fibrosis, and atherosclerosis. Importantly, studies show that changes in arterioles in response to hypercholesterolemia predate the formation of atherosclerotic plaques in large arteries [57]. Moreover, cardiovascular risk factors such as obesity and hypertension induce inflammatory responses at the level of arterioles [58,59,60,61,62]. In response to inflammatory cues, VSMCs acquire increased proliferative, migratory and invasive capabilities. Accordingly, inhibiting these dedifferentiation hallmarks would confer anti-inflammatory effects on arterioles.

In this study, we assessed the vasculoprotective role of MP against FBS-induced arteriolar SMC inflammation, a model mimicking mild arteriolar inflammation. Our results showed that MP, a novel homo-isoflavonoid that we isolated and characterized, inhibited FBS-induced proliferation and migration of human arteriolar smooth muscle cells. Moreover, MP attenuated VSMC adhesion and invasion as well as monocyte adhesion to VSMCs. This inhibition was concomitant with a decrease in matrix metalloproteases, MMP-2 and MMP-9, as well as an increase in the expression of myocardin, SM-22α, SM-α actin, calponin and caldesmon. On the other hand, MP decreased the expression of osteopontin, and abolished FBS-induced NF-κB expression and IĸB phosphorylation.

The significance of this study stems from three points: the novelty of the studied compound, the concentration used to conduct the experiments, and the relevance of the employed model. First, MP is a newly isolated and characterized homoisoflavonoid. Its potential vasculoprotective effects are established for the first time in this study. Moreover, MP proved to be potent at a sub-cytotoxic concentration. This may be promising especially in developing a noncytotoxic drug, which may have no or fewer side effects. To our knowledge, this is the first study using human arteriolar SMC as a model for arteriolar inflammation. Previous studies addressing vascular inflammation had used VSMCs extracted from large vessels. This is likely due to the challenging technical difficulty of isolating and maintaining a culture of microvascular smooth muscle cells isolated from human arterioles.

The origin of the VSMC greatly affect its response to various stimuli. For instance, studies reporting the effect of estrogen on VSMCs differ in different vascular beds. To address this, we recently published a paper where we elaborated on the effect of the vascular bed on various functional responses [63]. More relevantly, certain diseases such as retinopathies and kidney diseases affect arterioles rather than large vessels. Accordingly, microvascular SMCs would be a better model to recapitulate many aspects of these pathophysiological cues. However, whether MP effects mirror this vascular bed discrepancy remains to be established.

Overwhelming evidence shows that increased cell proliferation is a hallmark of VSMC phenotypic switch, especially in response to inflammation [64,65]. In our study, MP attenuated FBS-induced VSMC proliferation by blocking cells in the G_0_/G_1_ phase and inhibiting their progression to S phase. Progression from the G1 to S phase requires cyclin D1 [66,67]. Early in G1, cyclin D1 binds to CDK4. The resulting cyclin D1-CDK4 complex inhibits retinoblastoma protein, thus facilitating the transcription of S-phase genes [68]. Moreover, the activity of cyclin D1-CDK4 complex is inhibited by CDK inhibitors such as p21 and p27 [68]. Here, MP-induced cell cycle arrest was concomitant with an increase in the expression of CDK inhibitors, p21 and p27, as well as a decrease in both cyclinD1 and CDK4.

The anti-proliferative effect of MP is in accordance with the results of two previous studies. The first study reported that brazilin, a homoisoflavonoid, inhibited PDGF-induced proliferation of rat aortic VSMCs and induced cell-cycle arrest at G_0_/G_1_ phase [69]. The other study showed that the homoisoflavonoid-rich plant, *Ophiopogon japonicas*, exhibited anti-proliferative effects on thrombin-induced rat aortic VSMCs [70]. Similar to brazilin and *Ophiopogon japonicas*, MP induced the upregulation of p27 [69,70]. This suggests that homoisoflavonoids attenuate VSMC proliferation by targeting cell cycle regulators. Notably, p27 and p21 are regulated by ERK1/2 [71], which is a mitogenic factor itself [72]. Indeed, pharmacological [73] and genetic [47] inhibition of ERK1/2 inhibit VSMC proliferation. Here, MP attenuated FBS-induced phosphorylation of ERK1/2. This inhibition is in line with the effect of brazilin in VSMCs [69].

Clinically, arteriolar SMC proliferation has been previously assessed in two pathological conditions: hypertension [74] and menorrhagia [75]. VSMCs cultured from rat renal preglomerular arterioles showed that Angiotensin II significantly increased VSMC proliferation, indicating that VSMC hyperplasia may be associated with hypertension [74]. In menorrhagia, endometrial biopsies showed that proliferation of arteriolar SMCs varied between healthy and menorrhagic females [75]. Moreover, arterioles are responsive to pro-inflammatory cues [55], and thus are also remodeled during atherosclerosis by changing their cellular function and phenotype [55,57,76]. It is only reasonable to assume that inhibiting VSMC proliferation may aid in the ameliorating microvasculature in these pathological conditions. In this sense, MP, owing to anti-proliferative effect on arteriolar SMCs, presents a promising therapeutic potential at the level of microcirculation.

Many vascular complications arise from the imbalance in the proliferation/apoptosis ratio of VSMCs [77]. For example, VSMCs of diabetic patients have an increased level of the anti-apoptotic protein, Bcl2, and exhibit high proliferative rate [78]. This “failure to die” leads to alteration in the vessel microarchitecture [79]. In our study, MP-treated VSMCs became shrunk and translucent, with fine cytoplasmic extensions ending with blebs. All these features are characteristic of apoptotic cells [80]. These microscopic observations were confirmed by the fact that MP increased Bax/Bcl2 ratio and upregulated the level of activated caspase-3. Thus, MP induced apoptosis in FBS-induced micro VSMCs. It would thus be tempting to test whether MP can decrease thickening of muscularized arterioles in pulmonary artery hypertension or chronic obstructive lung disease [81,82].

Migration, invasion and adhesion of VSMCs are also major determinants of the dedifferentiated phenotype, and play a major role in vascular pathogenesis [83]. In our study, MP potently attenuated these hallmarks. Knowing that both ERK1/2 and FAK mediate cell migration [46,84], we may assume that MP-attenuated migration is achieved via inhibiting ERK1/2 and FAK phosphorylation. Moreover, in addition to its role in mobilizing adrenergic receptors to the cell surface [85], actin polymerization plays a key role in VSMC migration [52]. During migration, integrins are activated and clustered along with adhesion molecules at the migrating edge of the cell [86]. These cytoskeletal rearrangements along with actin polymerization facilitate cell crawling [87]. In our study, MP attenuated FBS-induced actin polymerization. Furthermore, MP reduced the invasive capacity of VSMCs by attenuating the expression of the matrix metalloprotease of MMP-2 and MMP-9. Previous studies report that VSMC migration was attenuated by the homoisoflavonoid, brazilin, and the homoisoflavonoid-rich plant, *Ophiopogon japonicas* [69,70]. However, these studies neither used human arteriolar cells nor assessed the effects on VSMC adhesion or invasion.

Clinically, arteriolar SMC migration was observed during cardiac transplantation in a condition termed transplant arteriosclerosis [88]. This condition is characterized by inflammation and intimal thickening due to the accumulation of SMCs from both donor and recipient [88]. A study using post-transplantation cardiac biopsy specimens from allograft patients showed that higher arteriolar SMC migration was associated with rejection grade, and thus with inflammation [88]. Here, MP attenuated arteriolar SMC migration. Hence, we may postulate that MP may contribute to reversing arteriolar inflammation and decreasing rejection grade.

Monocyte adhesion to blood vessels is a defining feature of vascular inflammation [89,90]. In response to inflammatory cues, arterioles become more permissive to monocytes, allowing them interact with endothelial cells and VSMCs [55]. Monocyte adhesion may also precipitate arteriolar barrier dysfunction [91]. Indeed, in response to increased luminal shear stress, monocytes are recruited to then adhere onto collateral arterioles [92]. Furthermore, direct evidence of monocyte adhesion to arterioles in response to Angiotensin II has been reported [93]. In our study, we showed that MP attenuated monocyte adhesion to PMA-activated arteriolar SMCs. Because PKC is a direct target of PMA [94], it may be postulated that MP attenuates PMA-provoked adhesion by inhibiting PKC. Indeed, PKC is inhibited by many flavonoids [95]. However, whether MP inhibits PKC is yet to be investigated.

VSMCs are not terminally differentiated, but are rather characterized by plasticity that allows phenotypic switch [96]. The extent of VSMC differentiation is determined by the expression level of differentiation markers. These markers are divided in to early, mid-term, and late differentiation markers, according to their order of appearance during embryogenesis [49]. The early differentiation markers include SM-α actin, myocardin, and SM22-α [49]. Caldesmon and calponin are considered to be mid-term differentiation markers [97]. Finally, desmin and smoothelin are among late differentiation markers [97]. Contractile VSMCs are characterized by the elevated expression of these markers [98]. In response to inflammation, VSMCs reduce the expression of these markers and adopt the expression of synthetic dedifferentiated phenotype [98]. VSMCs then become active in secreting ECM molecules such as osteopontin and osteonectin [51,99,100]. As such, high levels of these proteins indicate VSMC switch to synthetic phenotype.

Myocardin is a transcriptional co-activator that acts upstream of calponin and caldesmon. It is selectively expressed in cardiomyocytes and contractile SMCs [101,102,103]. It is a potent activator of the Serum Response Factor (SRF) where it stabilizes its binding at the CC(A/T-rich)_6_GG (CArG) cis-elements of CArG-dependent genes. Interestingly, expression of VSMC differentiation genes, such as SM22, MHC, SM α-actin and caldesmon requires CArG box in their promoter region and/or intronic sequences [104,105]. Nonetheless, some studies report that myocardin-driven expression of CArG-dependent SMC marker genes is not sufficient for the initiation of complete SMC differentiation [106].

In response to stimuli, myocardin regulation of SMC markers expression is attenuated mainly through 2 distinct pathways, both involving ERK1/2 activation. Activated ERK1/2 leads to Elk-1 phosphorylation, which competes with myocardin for SRF, leading to attenuated SMC marker expression [107]. Alternatively, myocardin may be directly phosphorylated by ERK1/2. This ERK1/2-induced myocardin phosphorylation hinders its ability to bind SRF and induce marker genes expression [108]. Several other effector pathways have been reported. For example the JAK/STAT signaling mediates VSMC switch to the synthetic phenotype via the interaction between STAT3 and myocardin [109]. Furthermore, Yap1 upregulation promotes synthetic phenotype by reducing myocardin-SRF interaction [110]. Similarly, NF-κB (p65) interacts with myocardin hindering myocardin-SRF interaction necessary for contractile genes expression [111]. Interestingly, myocardin attenuates the expression of NF-κB(p65)-driven genes [111]. In this sense, the protective role of myocardin lies in orchestrating the expression of SMC differentiation genes and in attenuating the expression of inflammatory genes.

Osteopontin is a glycoprotein secreted by many cell types including osteoblasts, monocytes, and VSMCs [112,113]. Specifically, its expression is increased during the switch of VSMCs to the synthetic phenotype as well as during vascular remodeling [114,115], as it is involved proliferation and migration [116,117,118]. Osteopontin induces migration by phosphorylating FAK, dephosphorylating downstream ILK, and by disrupting FAK-ILK interaction [118]. In addition, osteopontin leads to decreased expression of differentiation markers such as α-SM actin, and calponin [119]. Furthermore, studies show that osteopontin plays a role in vascular inflammation by inducing leukocyte chemotaxis and macrophage adhesion to endothelial cells [115]. Transcription of osteopontin is regulated by NF-κB [120,121].

Whereas previous reports have focused almost exclusively on assessing the expression level of these differentiation markers in macro-VSMCs [119,122,123], our study assessed the expression of these markers in micro-VSMCs. We showed that MP induced an elevation in myocardin, SM-α, SM-22α, calponin, and caldesmon, and reversed the FBS-attenuated expression of these markers in arteriolar SMCs. In fact, one previous study assessed the phenotypic switch of arteriolar SMCs in the context of benign nephrosclerosis (bN), a common hypertensive kidney damage characterized by fibrosis of renal arterioles [124]. Using renal tissue specimens, this study showed that arteriolar SMCs undergo a phenotypic switch in bN [124]. Surprisingly, their findings did not suggest an inverse correlation between caldesmon and dedifferentiated VSMCs [124]. Conversely, our results showed the caldesmon and calponin were upregulated by MP in FBS-induced arteriolar SMCs. Moreover, MP effectively decreased the expression of basal and FBS-induced expression osteopontin, further emphasizing the maintenance of a contractile phenotype of VSMCs. In 2018, Lin et al. showed that the flavonoid, (−)-epigallocatechin gallate (EGCG), attenuated Ag-II induced proliferation and migration in vitro and neointimal formation in vivo [125]. These inhibitory effects were mediated by myocardin. Therefore, this and other studies present myocardin as a molecular therapeutic target in vascular inflammation. In light of our results showing its ability to modulate myocardin expression, MP may thus be expected to possess a much-desired anti-inflammatory capacity. Indeed, our unpublished observations strongly suggest such an effect.

NF-κB is a member of the Rel-family of transcription factors [126]. Under normal physiological conditions, NF-κB is attached to its inhibitor protein IκB which traps it in the cytosol [126]. In response to inflammatory stimuli, IκB gets phosphorylated by its kinase, IκK, then ubiquitinated and degraded [122]. NF-κB then becomes free to translocate to the nucleus and trigger the transcription of pro-inflammatory genes [127,128]. In vascular inflammation, such as atherosclerosis, NF-κB upregulates the expression of adhesion molecules (ICAM-1and VCAM-1) and matrix-metalloproteases (MMP-2, and MMP-9) [129], further exacerbating vascular inflammation [130]. The vast majority of research show that NF-κB activates the inflammatory signaling in VSMCs of large vessels [127]. Except for one study showing that NF-κB is expressed and activated in arterioles [131], no previous studies assessed the modulation of NF-κB expression specifically in microVSMCs. Here, we show that NF-κB expression and IκB phosphorylation were attenuated by MP in FBS-activated micro VSMCs. This supports the vascular anti-inflammatory actions of MP and presents NF-κB and IκB as major molecular targets in the involved signaling pathway. By doing so, MP may be suppressing many genes such as MMP-2 and MMP-9, in addition to other genes which remain to be investigated.

## 5. Conclusions

**Figure 12:**
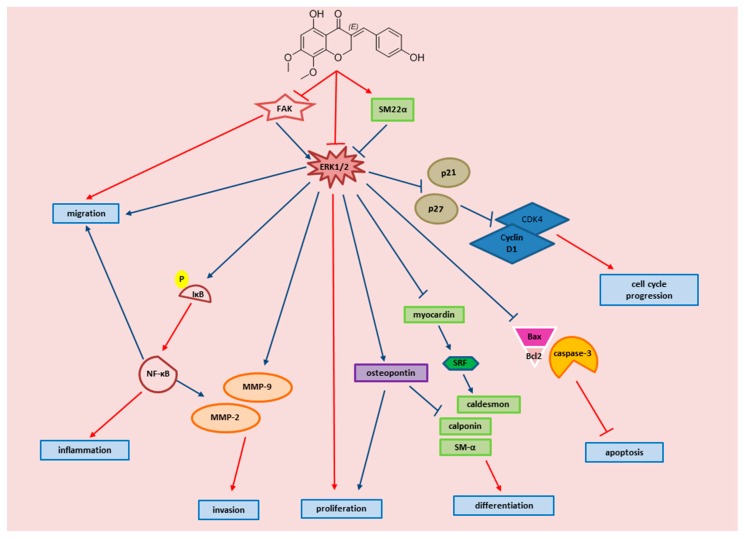
Schematic representation of the proposed signaling pathway by which MP attenuates FBS-induced inflammation of arteriolar *SMC.* The diagram displays key events of VSMC inflammation, and the molecular effectors by which MP attenuates these events. Red arrows are established by our study, blue arrows are established as referenced below: FAK → ERK1/2 [136,137]; SM22α → ERK1/2 [138]; ERk1/2 → osteopontin [139]; ERK1/2 → p21/p27 [71]; ERK1/2 → caspase-3, BAX/Bcl2 [140,141]; Osteopontin → proliferation [142]; Osteopontin → α SM and calponin [119]; p21, p27 → Cyclin D1/CDK4 [143,144]; NF-κB → MMP-2, MMP-9 [145,146,147]; NF-κB → proliferation, migration [148]; NF-κB → cyclin D1 [127,149].

To sum up, our data are consistent with the model shown in Figure 12, which illustrates different lines of action of MP and the involved molecular players. All depicted MP effects serve to attenuate VSMC dedifferentiation and thus may amend arteriolar inflammation. Further research is needed to better dissect the molecular mechanisms implicated in MP signaling. For instance, some flavonoids and isoflavonoids have been shown to regulate cAMP signaling in VSMCs [132,133]. Given the important and broad range of cAMP effects in human microvascular smooth muscle cells [32,34,86,134], if MP appears to modulate this pathway in these cells lines, one would expect to find diverse effects of MP on arteriolar physiology and pathophysiology. Further investigations are warranted to better determine the potential of MP as anti-inflammatory drug especially as pertains to vascular anti-inflammatory therapies. Knowing that modern drug therapy is still insufficient in preventing or treating CVD [135], the use of alternative medicine such as flavonoids and homoisoflavonoids may provide an important resource for potential new drugs.
